# Localized Electrical Impedance Myography of the Biceps Brachii Muscle during Different Levels of Isometric Contraction and Fatigue

**DOI:** 10.3390/s16040581

**Published:** 2016-04-22

**Authors:** Le Li, Henry Shin, Xiaoyan Li, Sheng Li, Ping Zhou

**Affiliations:** 1 Department of Rehabilitation Medicine, the First Affiliated Hospital, Sun Yat-sen University, Guangzhou 510080, China; Le.Li@uth.tmc.edu; 2 Department of Physical Medicine and Rehabilitation, University of Texas Health Science Center at Houston; TIRR Memorial Hermann Research Center, Houston, TX 77030, USA; Henry.Shin@uth.tmc.edu (H.S.); Xiaoyan.Li@uth.tmc.edu (X.L.); Sheng.Li@uth.tmc.edu (S.L.); 3 Guangdong Work Injury Rehabilitation Center, Guangzhou 510440, China

**Keywords:** electrical impedance myography, muscle contraction, fatigue

## Abstract

This study assessed changes in electrical impedance myography (EIM) at different levels of isometric muscle contraction as well as during exhaustive exercise at 60% maximum voluntary contraction (MVC) until task failure. The EIM was performed on the biceps brachii muscle of 19 healthy subjects. The results showed that there was a significant difference between the muscle resistance (R) measured during the isometric contraction and when the muscle was completely relaxed. *Post hoc* analysis shows that the resistance increased at higher contractions (both 60% MVC and MVC), however, there were no significant changes in muscle reactance (X) during the isometric contractions. The resistance also changed during different stages of the fatigue task and there were significant decreases from the beginning of the contraction to task failure as well as between task failure and post fatigue rest. Although our results demonstrated an increase in resistance during isometric contraction, the changes were within 10% of the baseline value. These changes might be related to the modest alterations in muscle architecture during a contraction. The decrease in resistance seen with muscle fatigue may be explained by an accumulation of metabolites in the muscle tissue.

## 1. Introduction

As a noninvasive bioimpedance-based technique, electrical impedance myography (EIM) is generally used to detect and quantify muscle health by sending high frequency, low intensity current into a discrete region of muscle tissue and measuring the consequent voltage [1]. Two basic EIM parameters include muscle resistance (R), which represents the resistivity to current flow through the intra- and extracellular fluids, and reactance (X), which indicates how the current flow is affected by cell membranes and by the various fascia of the body [2].

Most previous EIM studies have only dealt with measuring the muscle impedance while a muscle was relaxed, focusing on its architecture: the arrangement of the bundled fibers, the condition of the cell membranes, and the existence of connective and adipose tissue [3]. EIM has been most commonly used to reveal the anisotropy of healthy muscle as well as muscle disuse or atrophy [4,5,6]. Skeletal muscle is usually chosen as a focus because its bundled fiber structure facilitates current flow more readily along the fibers than across them [7]. Despite this, few EIM studies have investigated the changes in muscle impedance during a contraction. It is well known that the architecture and fiber geometry of the muscle changes during contraction [8,9]. Muscle imaging studies have proven that the pennation angle and muscle thickness changes during isometric contraction [10,11]. The resistance and reactance of a contracting muscle depends critically on the architectural or morphological factors of the muscle such as the changes in the size and shape as well as the shifting internal boundaries [12]. The impedance values also depend on the physiological factors of the muscle which include the complex conductive properties of the muscle medium itself at microscopic levels [13]. Shiffman and colleagues conducted impedance measurements on the anterior forearm with isometric contractions of the finger muscles [13]. Their data showed an increase of muscle resistance and reactance during the grasp. However, this was a case study and the measurement was completed without a systematic control of contraction levels and the impedance was not localized to the specific muscle related to the grasp. More recently, animal models have been utilized to study EIM changes during passive muscle contraction caused by electrical stimulation [14]. The results also reported an increase of resistance with an increase in stimulus intensity.

Another understudied area of EIM is its relation to changes in muscle during a fatigue state. Muscle fatigue has been defined bio-mechanically as an inability to maintain an expected force or power output [15] and is a symptom in a wide range of pathological conditions including various neurological disorders and neuromuscular diseases [16,17]. A previous study has shown that muscle activity gradually increases during sustained isometric muscle contractions [18,19]. Benson and colleagues also found blood lactate levels were increased in the biceps brachii following an exhaustive exercise [19]. In addition, muscle acidity was shown to change with a reduction in net lactate production rate after exhaustive exercise [20]. The connection between these various changes to the underlying muscle physiology and its impedance has not been well investigated.

Among the various methods of EIM measurement, hand-held electrical arrays (HEA) provide a convenient way for measuring muscle impedance. The multi-frequency measurements obtained with the HEA have also demonstrated very high test-retest reproducibility [21]. However, the HEA has not been applied to investigate the impedance of a contracting muscle nor impedance changes in fatigued muscles. In both of these types of studies, electromyogram (EMG) analysis is most commonly involved, but is limited in EIM data. Accordingly, the objective of the current study was to apply HEA and assess whether muscle impedance varies during different levels of isometric contraction. The local changes in EIM parameters were also examined during a sustained contraction held until task failure.

## 2. Methods

### 2.1. Subjects

Nineteen healthy subjects (11 female and eight male, age 35 ± 10 years, range from 23 to 46 years) participated in this study. The subjects had no known history of neurological disorders or neuromuscular disease and had normal strength and bulk of the biceps brachii muscle. The study was approved by the Institutional Review Board of University of Texas Health Science Center at Houston and the TIRR Memorial Hermann Hospital (Houston, TX, USA). All the subjects gave their written consent before the experiment procedures.

### 2.2. Experiment

**Figure 1:**
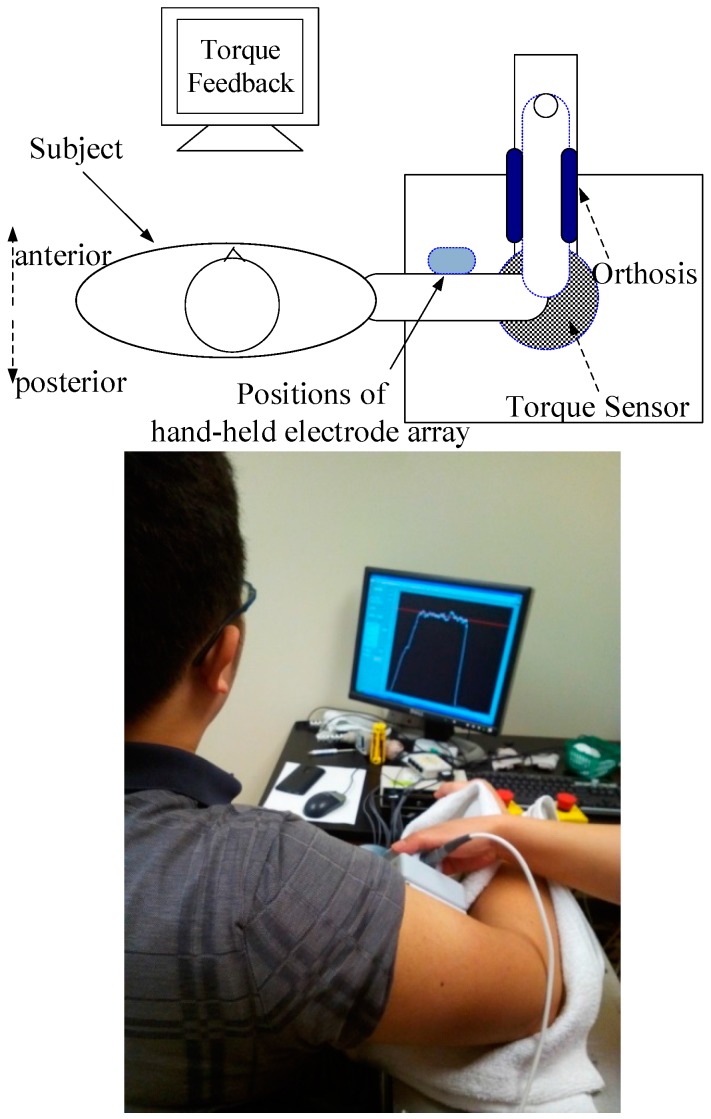
Experimental setup of EIM measurement during contraction.

The experiments were performed on the biceps brachii muscle of the dominant arm. The subjects were seated in a height-adjustable chair with the examined arm secured firmly on a customized elbow torque apparatus with elbow joint at 90° flexion and the shoulder at 45° abduction. The wrist joint was supported and put in the neutral position (Figure 1). Two pairs of vertical plates at the proximal and distal forearm were used for stabilization [22]. The laboratory maintained a constant temperature (approximately 71 °F/22 °C) during all the experiments.

Impedance measurements were made using a handheld electrode array system (EIM1103, Skulpt Inc., Boston, MA, USA) which applies very low-intensity, high-frequency electrical currents at frequencies ranging from 1 kHz to 10 MHz, and then measures the consequent surface voltages [21]. The sensor array was placed over the center of the muscle belly. The distance between the current bar electrodes (3.9 cm long, 0.4 cm width) is 4.5 cm, and between the voltage bar electrodes (1.3 cm long, 0.4 cm width) is 1.7 cm [23]. The electrode placement was longitudinal with the muscle fibers. A marker pen was used to ensure the sensor was at the same position in each measurement. A contact check was performed automatically by the EIM system before each data collection and sterile saline wipes (Hygea, PDI Inc., Orangeburg, NY, USA) were applied to ensure the skin was sufficiently moist prior to performing impedance measurements.

Impedance measurements were performed during each of the following four isometric contraction conditions: (1) baseline value at rest; (2) the MVC condition; (3) low level contraction at 20% MVC; and (4) high level contraction at 60% MVC [24]. Torque during isometric contraction was recorded via a torque sensor (Model TRS 500, Transducers Techniques, Temecula, CA, USA) and displayed on a computer monitor in front of the subject. For the MVC condition, subjects were asked to perform maximal effort of elbow flexion against the torque apparatus. Subjects received strong verbal encouragement during three MVC attempts. The trial with the greatest torque value was used to determine the MVC value. For the low and high level contractions, the subjects were given a target on the screen and asked to hold and match their torque to the target line. The target was set to the corresponding percentage level of the subjects’ maximal effort. Each contraction lasted for approximately 5 s which was sufficient to complete the impedance measurement. Three trials were conducted at each contraction level and there was at least 2 min of required rest between each trial. The order of steps (3) and (4) was randomized for each subject.

The subject was given at least a 10 min break before the second part of the experiment. For the fatigue contraction test, the subjects were in the same torque apparatus and the target force level on the computer display was set to 60% MVC [25]. They were asked to maintain isometric contraction at this level for as long as possible. The EIM measurement was measured repeatedly in succession throughout the sustained contraction task (each EIM measurement took about 4 s) until task failure. The criterion for task failure was set as the inability to the keep the force within 10% of the target level for >3 s, despite strong verbal encouragement [24]. The time to task failure was also noted. EIM values were examined at the following four conditions: (1) during the rest condition right before fatigue task start (baseline); (2) immediately after the start of the contraction; (3) at the end of the contraction (*i.e.*, the last measurement right before task failure); and (4) post fatigue at rest after 1 min.

### 2.3. Data Analysis and Statistics

The resistance (R) and reactance (X) (mean ± standard deviation) were analyzed and reported. X, R *vs.* applied frequency plots were also generated. Resistance and reactance obtained at 50 kHz and 100 kHz frequencies were used to examine the effects of muscle contraction and fatigue. The frequency selection of 50 kHz is based on its common usage among other commercially available electrical impedance devices and 100 kHz was chosen as a higher frequency which is still within the optimal frequency band and could additionally study the anisotropy of the muscle [26]. Two-way repeated ANOVA was used to compare the resistance and reactance at different contraction levels (*i.e.*, Rest, 20% MVC, 60% MVC, MVC), as well as different conditions in the fatigue test (*i.e.*, baseline before fatigue task, at the beginning of the contraction, right before task failure, and post fatigue rest) (between group factor as 50 kHz and 100 kHz, within group as contraction states (Levels = 4) for both experiments). The Bonferroni correction was used in pairwise comparison in the post-hoc tests. Pearson correlation analysis was conducted between the changes of resistance during sustained fatigue test and the time to task failure. Statistics analysis was done using SPSS17 (IBM Inc, Seattle, WA, USA). The significance level was determined as *p* < 0.05 for all statistical analyses.

## 3. Results

**Figure 2:**
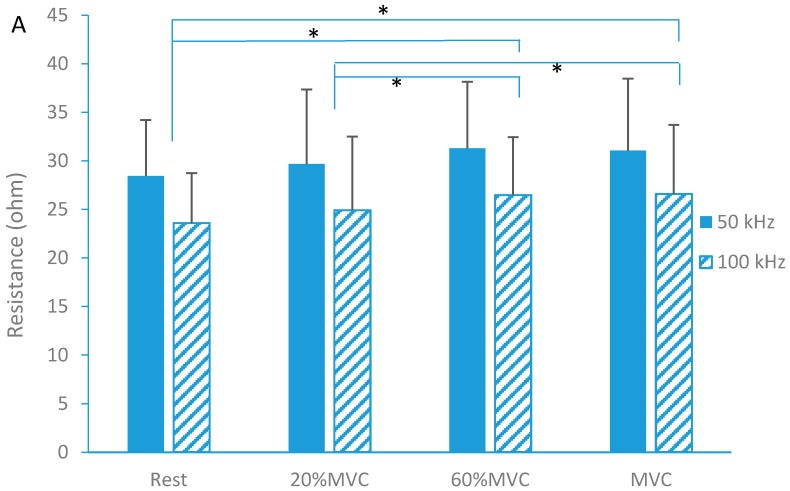

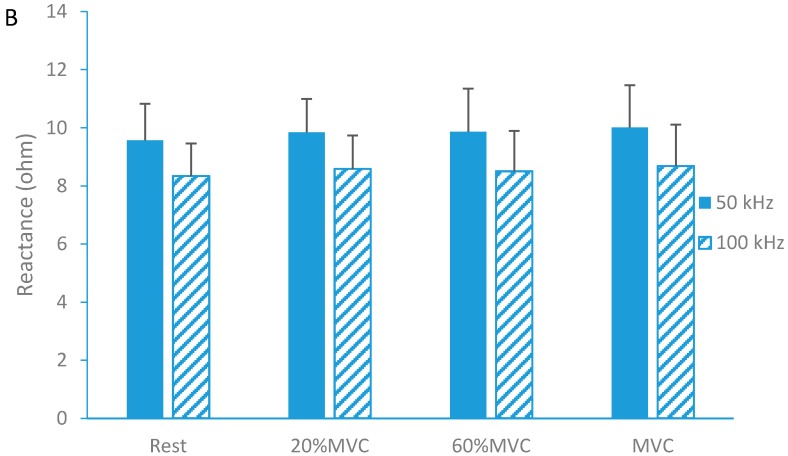
Resistance (**A**) and Reactance (**B**) at isometric contraction with two different frequencies (50 kHz and 100 kHz) (mean ± standard deviation, * indicates *p* < 0.05 between contraction levels).

The two-way ANOVA results showed a main effect of isometric contraction on resistance (R) (F = 17.42, *p* < 0.001). *Post hoc* analysis showed that R increased 10.1% (*post hoc* test, *p* < 0.001) and 9.2% (*post hoc* test, *p* < 0.001) at the high level contractions (60% MVC and MVC, respectively) as compared to the rest (Figure 2A).

In addition, there were also significant differences in R between the lower contraction (20% MVC) and higher contraction levels (5.5% increase at 60% MVC with *p* = 0.005 and 4.6% increase at MVC with *p* = 0.026, respectively). However, *post hoc* tests did not reveal significant changes of resistance between rest and lower contraction (20%MVC) (*p* = 0.199). Between-group comparison showed R at 50 kHz (30.12 ± 1.57 Ω) was significantly larger than R at 100 kHz (25.39 ± 1.44 Ω). In contrast to R, there were no significant changes in reactance (X) during isometric contraction (Figure 2B).

**Figure 3:**
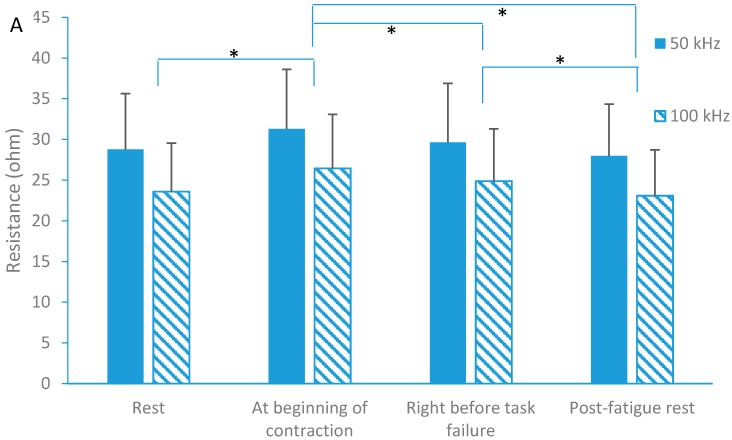

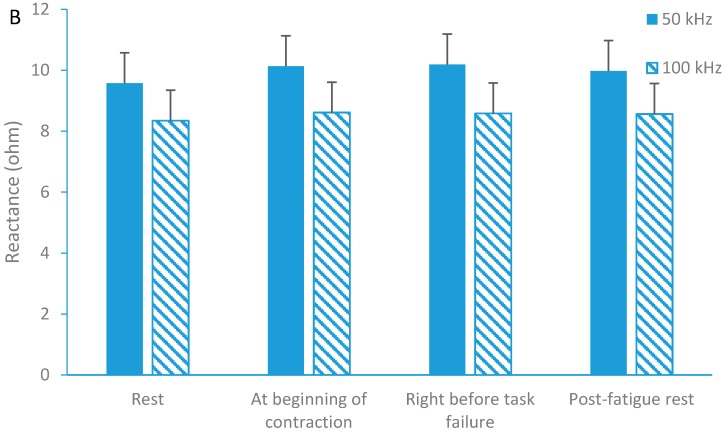
Resistance (**A**) and Reactance (**B**) at fatigue tests with two different frequencies (50 kHz and 100 kHz) (mean ± standard deviation, * indicates *p* < 0.05 between contraction conditions during fatigue task).

For the fatigue effects, the results showed that the resistance (R) changed significantly during the four different testing conditions (*p* < 0.001; F = 19.959). The resistance decreased right before task failure compared to the beginning of contraction(post hoc test, *p* = 0.014), and post fatigue resistance further decreased as compared to right before task failure(post hoc test, *p* = 0.011) (Figure 3).

**Figure 4:**
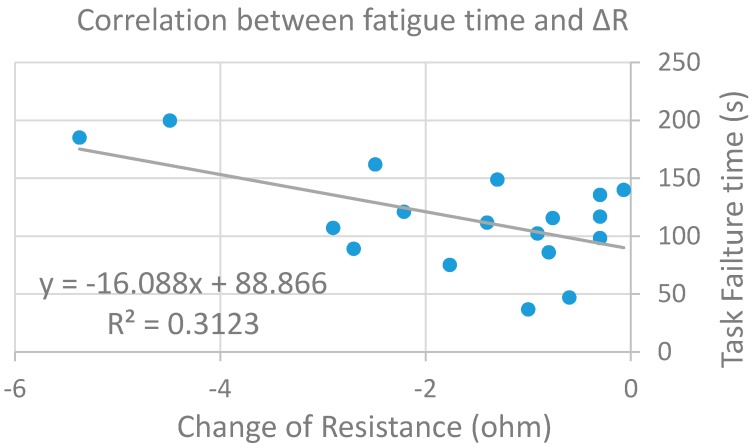
Correlation between the task failure time and the change of resistance during sustained contraction in fatigue test (*p* = 0.0159).

However, there was no significant difference in resistance between baseline and post fatigue rest (*p* = 0.143). The between-group comparison showed that the resistance at 50 kHz (29.42 ± 1.57 Ω) was significantly larger than that (24.50 ± 1.39 Ω) at 100 kHz. For reactance (X), there was no significant difference during fatigue process (*p* > 0.05). Correlation analysis revealed a significant relationship (*p* = 0.016) between the time to task failure and the decrease of resistance (changes immediately after the start of contraction to right before task failure) with R^2^ = 0.3123 (Figure 4).

## 4. Discussion

In this study, we investigated electrical impedance myography during different levels of isometric muscle contraction as well as during a fatigue task. We found there was a significant increase in resistance during high level contractions and a decrease in resistance with muscle fatigue. Such changes might be related to architectural and metabolic changes which occur in contracting muscle. Our results revealed a significant increase of resistance (R) between the muscle at rest to a 60% MVC contraction, but a decrease to the baseline after fatigue. This suggests that R reflects a net resistance from both architectural and metabolic changes in contracting muscle. This was further supported by the finding of a continued decrease in R post-fatigue. The increase in resistance is in line with several previous findings from human subjects and animal models.

Shiffman and colleagues found an increase of resistance and reactance with a linear setup of EIM measured on the upper limb as hand grip forces were generated and increased [13]. Rutkove also revealed a small increase of resistance in the order of a few percent of the baseline value [1]. In a rat hindlimb model, Sanchez *et al.* reported that the passive contraction of muscle induced by electrical stimulation also elevated the resistance of the muscle [14]. Shiffman *et al.* suggested that these changes in impedance associated with muscle contraction might be related to twofold effects [13]. First, shifts in the internal morphology of muscle tissues and the relative movements of the insulating boundaries (connective tissues) may act to obstruct current flow which then increases the resistance [12,13]. Second, with gross morphological increases in muscle cross-section area during contraction, the “easy pass” medium under the sensor for the current path going through the arm is enlarged which in turn decreases the impedance since muscle tissue is much more conductive than subcutaneous fat and connective tissues [11]. These two factors (conflicting with each other) might explain the small percentage increase of R in our results. However, the increase of R does not rule out the fundamental electrophysiological changes (*i.e.*, metabolic state) in the tissue occurring during contraction which might be simply overwhelmed by the morphological effects of the fiber and boundary rearrangements. For example, by applying a 5-element resistor-capacitor model and assessing R and X during muscle contraction, Kashuri and colleagues found the extracellular fluid resistance increased around 3.9%, while the membrane capacitance increased around 5.6% at the forearm when the finger flexor generated a 10 kg force lasting 8–10 s [27]. As suggested by Rutkove, the effects of muscle excitation and contraction on EIM merits caution since the results can vary depending on the location of the recording electrodes on particular muscle, as well as the configuration of the injecting and measuring electrodes [1]. Our localized EIM on biceps brachii during isometric contraction added the evidence of increasing resistance in a small percentage when muscle generates a higher force.

Reactance is modeled as related to the obstruction to current flow produced by the presence of capacitors [12]. Shiffman and colleagues found increases in reactance during contraction and the changes in the reactance can arise via changes in the intracellular resistors of the network and from the cell membranes when capacitive effects occur [13]. In this study, we did not find significant reactance changes during isometric contraction. The discrepancy might come from a different setup of the electrodes and measured positions. The different trend of change between resistance and reactance are also related to inherent electrical properties, the conductivity and permittivity of the muscle, subcutaneous fat tissue and connective tissue [28]. Garmirian and colleagues revealed that the muscle impedance was impacted by the electrical conduction of muscle which is related to the properties of individual fibers, in particular via the capacitance of membranes and the arrangement of the fibers in columnar order [26]. By using a rat hindlimb suspension model to study muscle disuse, Li *et al.* found that a reduction in muscle fiber size reduces the charge storage capacity of the tissue which in turn revealed that membrane capacitance had the greatest impact on reactance values [6]. The contraction does not change the fiber size itself, thus not contributing much to the changes of membrane capacitance.

Fatigue is a complicated psychophysiological state which could be described as a feeling of weakness or muscle pain, or a decrease in force performance [29]. In the sustained 60% MVC contraction (held to task failure), fatigue was obvious to our subjects, demonstrated as visible muscle tremors when they struggled to hold the level constantly after approximately 1 min of contraction. We found significantly decreased resistance with muscle fatigue, which might be due to the accumulation of metabolites and intracellular fluids during muscle contraction which may improve the conductivity of the muscle. Thus, the resistance right before task failure was lower compared to beginning of contraction. Allen and colleagues’ review on skeletal muscle fatigue supported the idea that increased metabolites during sustained isometric contraction contributed to fatigue [30]. In order to further verify the potential relationship between the duration of sustained contraction until fatigue and the increase of metabolite accumulation, we did a correlation analysis between the time to task failure and the change of resistance. The results showed a significant positive correlation between the change of resistance and the contraction time. A previous study on muscle circulation and fatigue in healthy control subjects showed that muscle ischemia occurs during sustained isometric contractions [31]. Another study revealed that fatigue continues to develop throughout the contraction and if a muscle is stimulated continuously at a frequency close to that producing the maximal force, then force production generally shows a rapid decline often called high-frequency fatigue [32]. A characteristic of this type of fatigue (which is similar to the protocol in the current study) is that the recovery is also very rapid, often having a time course of recovery of only 1–2 s [33]. Similarly, Taylor and Gandevia also found that fatigue starts to remit when the task is complete [34]. This may explain our finding of no significant difference between the baseline and post-fatigue resistance since the muscle may have recovered in the rest period. In regards to the reactance, there was no significant difference found due to fatigue. Similarly, Aaron and Shiffman revealed that reactance (but not the resistance) returned quickly to its baseline after the dynamometer was released at the end of a sustained section, even though the muscle might be in fatigue state [12]. The resistance changes without reactance alterations during muscle contraction may add further support to suggest that metabolite accumulation is the main effect on impedance while the membrane properties may not dramatically contribute to the muscle impedance alterations after sustained contractions. As the muscle fatigue may come from both central and peripheral factors, a combination of EMG together with EIM can be used to further investigate fatigue-related neuromuscular changes.

The reactance of a membrane depends on the frequency of the applied current, and consequently, so does the measured impedance [2]. Recent work on the frequency dependence of ΔR and ΔX under isometric contraction showed an even stronger relation to biochemical changes, in that parameters describing the state of the muscle fibers grew in qualitative agreement with the force-time integral [35,36]. Adjusting the frequency of the applied current shifts the relative weights of resistive (intra- and extracellular fluid) and reactive (cell membrane) contributions to the muscle impedance, and actually at sufficiently high frequencies the cell membranes make essentially no contribution to impedance at all [1,12]. Similarly in our study, we found the 100 kHz caused lower resistance and reactance compared to 50 kHz. Shiffman and colleagues (2008) also revealed that in a “3 element circuit” model when the current frequency is very high, the resistance is approximate to a constant and the reactance is inversely proportional to *f* [36]. Although the multi-frequency capabilities are an advantage of the EIM system, this current first study only focused on the comparison of muscle EIM changes with contraction states. It would be interesting to have a frequency spectrum analysis with the contraction changes, which might be useful in the clinical setting. The frequency at which the phase is maximal may provide a useful diagnostic tool [37,38,39].

Healthy subjects between the ages of 23 and 46 were recruited in this study. For each subject the EIM changes at different conditions were examined. Previous studies have shown that aged muscle has significant difference compared to younger groups [40,41]. Therefore, further study is warranted to compare the changes of EIM parameters during isometric contraction in young and aged muscles. In addition, we only considered muscle contraction in one direction (longitudinal) with the measurement of EIM data, while rotation of the electrode may result in a different measurement which is more useful to study the anisotropy of muscle [4]. It would be interesting to check the electrical anisotropy of the contracted muscles in a future study. Another point to consider when interpreting the results in this current study is that the changes of electrical impedance may come from confounding factors in the nature of the conducting medium as well as in geometrical changes. It is difficult to attribute the changes to any specific factor. Combining EIM with another imaging technique, such as ultrasound, might be a possible way to further explain the electrical impedance changes. For example, we only found the resistance changes at higher level of muscle contraction (*i.e.*, >60% MVC) but not the lower level (*i.e.*, 20% MVC), and morphology information from ultrasound might provide further useful information. Finally, although this technical study only examined healthy subjects, the findings can provide useful information for clinical application of the EIM. During examination or tracking of neuromuscular diseases using EIM, the effect of muscle contraction and fatigue on EIM parameters should be considered for data analysis and interpretation if the measurement is not performed on a resting muscle.

In summary, our results demonstrated there was a significant increase in resistance during isometric contraction but the changes were not dramatic compared to the baseline value (at rest). The resistance changes might be related to the compound effects of muscle architectural and physiological factors. In addition, the impedance data appeared to plateau at the higher contraction levels as there were little changes above 60% MVC. Our fatigue experiment demonstrated a decrease of resistance which may be induced from the accumulation of metabolites attributed to the fatigue state. There were no significant changes in reactance during neither isometric contraction nor fatigue. These impedance-based measures in different muscle contraction situations add important insights for analysis and interpretation of EIM parameters. These insights may be helpful when further extended to clinical evaluations of changes in force generation in patients with neurological disease.
